# A facile approach to hydrophilic oxidized fullerenes and their derivatives as cytotoxic agents and supports for nanobiocatalytic systems

**DOI:** 10.1038/s41598-020-65117-7

**Published:** 2020-05-19

**Authors:** Panagiota Zygouri, Konstantinos Spyrou, Efstratia Mitsari, María Barrio, Roberto Macovez, Michaela Patila, Haralambos Stamatis, Ioannis I. Verginadis, Anastasia P. Velalopoulou, Angelos M. Evangelou, Zili Sideratou, Dimitrios Gournis, Petra Rudolf

**Affiliations:** 1 Department of Materials Science and Engineering, University of Ioannina, GR-45110, Ioannina, Greece; 2 Zernike Institute for Advanced Materials, University of Groningen, Nijenborgh 4, NL-9747AG, Groningen, the Netherlands; 3 Grup de Caracterització de Materials, Departament de Física and Barcelona Research Center in Multiscale Science and Engineering, Universitat Politècnica de Catalunya, EEBE, Campus Diagonal-Besòs, Av. Eduard Maristany 10-14, 08019 Barcelona, Spain; 4 Department of Biological Applications and Technologies, University of Ioannina, GR-45110, Ioannina, Greece; 5 Laboratory of Physiology, School of Medicine, Faculty of Health Sciences, University of Ioannina, GR-45110, Ioannina, Greece; 6 Institute of Nanoscience and Nanotechnology, NCSR “Demokritos”, 15310 Aghia Paraskevi, Attikis, Greece; 7 Present Address: Department of Radiation Oncology, The Perelman School of Medicine, University of Pennsylvania, 3400 Civic Boulevard, Philadelphia, PA 19104 USA

**Keywords:** Biotechnology, Carbon nanotubes and fullerenes

## Abstract

A facile, environment-friendly, versatile and reproducible approach to the successful oxidation of fullerenes (oxC_60_) and the formation of highly hydrophilic fullerene derivatives is introduced. This synthesis relies on the widely known Staudenmaier’s method for the oxidation of graphite, to produce both epoxy and hydroxy groups on the surface of fullerenes (C_60_) and thereby improve the solubility of the fullerene in polar solvents (e.g. water). The presence of epoxy groups allows for further functionalization *via* nucleophilic substitution reactions to generate new fullerene derivatives, which can potentially lead to a wealth of applications in the areas of medicine, biology, and composite materials. In order to justify the potential of oxidized C_60_ derivatives for bio-applications, we investigated their cytotoxicity *in vitro* as well as their utilization as support in biocatalysis applications, taking the immobilization of laccase for the decolorization of synthetic industrial dyes as a trial case.

## Introduction

The discovery^1^ of fullerene (C_60_) in 1985 and the establishment of a protocol for its bulk production^2^ has had a widespread impact throughout science due to C_60_’s special physico-chemical and optical properties, as well as the specific chemical reactivity resulting from the unique cage structure^3^. Fullerene research has produced breakthrough highlights in the fields of superconductivity, organic ferromagnets, photovoltaics, thin-film transistors, and catalysis^4–13^. Nonetheless, the exploitation of this extraordinary molecule for applications in disciplines such as biochemistry, biology and medicine is hampered by its insolubility in a large number of solvents including especially water, where aggregation of the C_60_ molecules into micelle-like clusters is observed^14^. This problem has been addressed with the help of functionalization chemistry^15,16^, leading to water-soluble fullerene hybrids^17,18^ and to the synthesis of numerous fullerenes derivatives^19,20^ targeted to meet specific needs in materials science^21^, biomedical chemistry^18,22–24^, and pharmaceutical research^25^.

Over the last decades covalent functionalization of fullerenes has been extended to include various functionalization reactions^3,19,25–27^, among which the most commonly employed is the Prato reaction for fullerene functionalization through fulleropyrrolidine formation based on the 1,3 dipolar cycloaddition^28–30^. As a result, a plethora of organic reagents rich in biological and pharmaceutical activity have been covalently attached to C_60_, yielding derivatives with enhanced properties for a broad range of biological and medicinal applications^18,23,31–36^. All these reactions imply complicated manipulation and require special experience in handling. There is therefore a growing demand for controllable and easy-to-handle methods for the functionalization of C_60_.

Here we report a novel, easy, versatile, and reproducible procedure for the chemical oxidation of C_60_, on the basis of Staudenmaier’s method^37^, which is a controllable synthesis, extensively studied for the oxidation of graphite into graphene oxide^38^. We wish to emphasize that we used a variant of Staudenmaier’s method here because fullerenes are more sensitive than other carbon forms (CNTs, Graphene) and hence direct application of Staudenmaier’s method may lead to insufficient and not well-defined structures and functional groups. We show that through a variant of this method, the properties of pristine fullerenes can be tailored, introducing a combination of oxygenated functional groups, which in turn constitute reacting sites for chemical derivatization. In comparison with the other oxidation methods^39–49^ our proposed procedure exhibits a higher yield of oxygen functional groups and therefore greatly enhances the fullerene’s hydrophilicity, while the epoxy groups provide the ability to interact covalently with amines at ambient conditions. Contrary to other protocols reported in the literature*,* this synthesis method does not require high temperatures^50^ nor does it lead to the creation of clusters, aggregation or by-products^51^, all phenomena hindering hydrophilicity^52^. Our approach is suitable for up-scaling to mass production because, differently from other methods reported, because it presents no inherent synthetic difficulties and/or yield limitations^51,53,54^. In more detail, as a consequence of the strong acid treatment, the surface of the fullerene molecule is decorated with diverse oxygen functionalities, converting the completely insoluble fullerene into a hydrophilic molecule soluble in many polar solvents, while maintaining its stereochemistry (spherical shape). To substantiate this claim, we have investigated the evaluation of the *in vitro* cytotoxic activity of the C_60_ hybrids, performed against mouse leiomyosarcoma (LMS) and human lung cancer (A549), as well as a normal cell line, normal human fetal lung fibroblasts (MRC-5).

An additional huge benefit derived from the creation of epoxy moieties is the possibility of further functionalization with numerous organic species via covalent bonding to the epoxy groups, a method that has been extensively employed for the chemical functionalization of graphite oxide^55–57^. Further functionalization of oxidized C_60_ with a primary aliphatic amine was performed to confirm the presence of epoxy moieties and to attest this oxidative method as a controllable and reproducible step for the creation of new C_60_ derivatives. X-ray photoelectron (XPS), Raman and Fourier transform infrared (FTIR) spectroscopies, differential scanning calorimetry (DSC) and thermogravimetric analysis (TGA), in conjunction with powder X-ray diffraction (XRD) measurements were performed for the material’s characterization.

**Figure 1:**
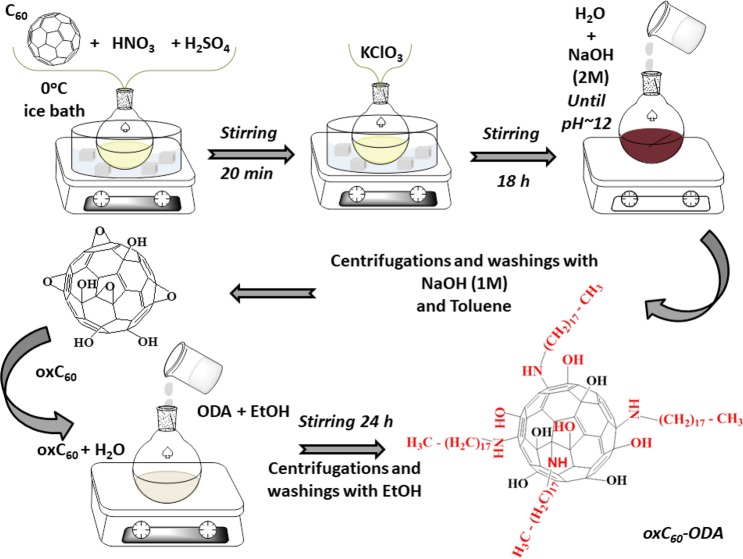
Synthetic method for the production of oxidized C_60_ and its derivatives.

The following figure (Fig. 1) illustrates the synthetic method for the production of oxidized C_60_ and its derivatives.

Laccases (benzenediol: oxygen oxidoreductase; E.C. 1.10.3.2) belong to multi-copper containing oxidases and their activity is based on the reduction of molecular oxygen to water with the simultaneous one-electron oxidation of aromatic substrates^58^. In addition, laccases, due to their ability to oxidize different substrates, are potential candidates for many biotechnological and industrial applications, such as decolorization of synthetic dyes^59^. Herein, the immobilization of a native laccase from White rot fungi on the newly synthesized oxC_60_-ODA was performed and further used for the decolorization of two synthetic dyes with industrial applications.

## Material and methods

### Production of oxidized fullerene (oxC_60_)

**oxC**_**60**_ was obtained by a modified Staudenmaier’s method. In a conical flask, 100 mg of C_60_ (98%, Sigma-Aldrich) were added upon stirring to a mixture of 4 mL of H_2_SO_4_ (95–97%) and 2 mL of HNO_3_ (65%) kept in an ice-water bath at 0 °C. 700 mg of KClO_3_ were then added in little portions to the solution under vigorous stirring and the reaction was continued for 18 hours. 20 mL water were then added and the mixture was stirred for another 30 minutes. The resulting aqueous acid mixture was neutralized by slowly adding a 2 M NaOH solution until a pH of 12 was reached. The precipitate was separated from the solution by centrifugation, washed with a 1 M NaOH solution and centrifuged again^60^. The product was washed three times in order to eliminate the remaining salts used or formed during the oxidation procedure. The precipitate was dispersed in 50 mL of toluene, stirred for 20 min and centrifuged to remove unreacted fullerenes. Finally, the oxidation product was dispersed in ethanol and air-dried on a glass plate. The final material possessed a yield of approximately 50% (based on the weighted mass).

### Characterization tools

Fourier transform infrared (FT-IR) spectra over the spectra range 400–4000 cm^−1^ were recorded with a Perkin–Elmer Spectrum GX infrared spectrometer featuring a deuterated triglycine sulphate (DTGS) detector. Every spectrum was the average of 64 scans taken with 2 cm^−1^ resolution. Samples were prepared as KBr pellets with ca. 2 wt% of sample. Raman spectra were collected with a Micro – Raman system RM 1000 RENISHAW, using a laser excitation line at 532 nm (Nd – YAG), in the range of 1000–2400 cm^−1^. A power of 1 mW was utilized with a 1 μm focusing spot so to avoid photodecomposition of the samples. ^13^C NMR spectrum of oxC_60_ was recorded in D_2_O using a Bruker Avance DRX spectrometer operating at 125.1 MHz. Thermogravimetric measurements were carried out with a Perkin Elmer Pyris Diamond TG/DTA. Samples of about 5 mg were heated in air from 25 °C to 900 °C, at a rate of 5 °C min^−1^. Differential scanning calorimetry was applied using a Q100 Thermal Analysis instrument between 25 °C and 200 °C under inert atmosphere (nitrogen), at heating and cooling rates of 2 °C min^−1^. DSC measurements were done placing the sample in an open vessel to reproduce the conditions of TGA. High-resolution X-ray powder diffraction (XRD) patterns were collected with a monochromatic Cu K_α_ (λ = 1.5418 Å) radiation source (operated at 30 keV and 35 mA) and a vertically mounted INEL cylindrical position-sensitive detector (CPS120), used in the Debye-Scherrer geometry to enable simultaneous acquisition of the diffraction profile over a 2θ range between 4° and 120° (with an angular step of 0.029° (2θ)). For the XRD measurements, the powder was placed into a Lindemann capillary tube (0.5 mm diameter). X-ray photoelectron spectroscopy (XPS) measurements were performed under ultra-high vacuum (2 ×10^−9^ mbr) and data were collected using an SSX-100 (Surface Science Instruments) instrument equipped with a monochromatic Al K_α_ X-ray source (hν = 1486.6 eV). The energy resolution was set to 1.2 eV in order to minimize data acquisition time and maximize the signal-to-noise ratio and the photoelectron take off angle was 37° with respect to the surface normal in order to minimize data acquisition time and maximize the signal-to-noise ratio. All binding energies were referenced to the C1*s* core level photoemission line at 285.0 eV^61^. Spectral analysis included a Shirley background subtraction and peak deconvolution with Gaussian-Lorentzian functions in a least-squares curve-fitting program (Winspec) developed at the Laboratoire Interdisciplinaire de Spectroscopie Electronique, Universitaires Notre-Dame de la Paix, Namur, Belgium. For the N1*s* line, we applied a linear background subtraction because the low peak intensity did not allow for Shirley background subtraction. The photoemission peak areas of each element used to calculate the amount of each species within the probed volume were normalized to the sensitivity factors of each element specific to the spectrometer. All the measurements were made on freshly prepared samples in order to assure the reproducibility of the data. Three different spots were measured on each sample to check for reproducibility.

### Evaluation of the *in vitro* cytotoxic activity of oxC_60_ - Cell lines

To consistently evaluate the cytotoxicity of water-soluble oxC_60_ we used two cancer cell lines, mouse leiomyosarcoma (LMS) and human lung cancer (A549) as well as a normal cell line, normal human fetal lung fibroblasts (MRC-5). The latter was kindly provided by Dr. Evangelos Kolettas, Laboratory of Biology, School of Medicine, Faculty of Health Sciences, University of Ioannina, Greece. All different cell lines were cultured in Dulbecco’s Modified Eagles Medium (DMEM) enriched with 10% fetal bovine serum (FBS), 100 IU/mL penicillin, 100 μg mL^−1^ streptomycin and 1.4 mM L-Gloutamin, at 37 °C, with 5% CO_2_. MTT assay: The ability of the oxidized fullerenes to inhibit the cell growth was expressed by the average IC_50_ value (oxC_60_ concentration required for 50% inhibition of cell growth) and was analyzed using the MTT assay (3-(4,5-dimethylthiazol-2-yl)-2,5-diphenyltetrazolium bromide)^62^. Briefly, 3 × 10^3^ for both LMS and A549 cells, as well as 5 × 10^3^ MRC-5 cells, were cultured overnight on 96-well plates and culture media containing different concentrations (ranging from 200 to 2000 μg mL^−1^) of oxC_60_ were added. The oxC_60_ was dissolved in sterilized water (solvent). The 96-well plates with culture media containing different volumes of solvent, equal to volumes of solutions added to the test wells, were considered as control. After incubation for 48 h, 50 μL of MTT were added in each well from a stock solution (3 μg mL^−1^), and incubated for additional 3 h. The yielded purple formazans were re-suspended in 200 μL of DMSO, using a multi-channel pipette. The solution was spectrophotometrically measured (540 nm, subtract background absorbance measured at 690 nm) using a microplate spectrophotometer (Multiskan Spectrum, Thermo Fisher Scientific, Waltham, USA). All the experiments were performed at least in triplicate. IC_50_ values were determined by the curve of percentage of inhibition versus dose.

### Further chemical functionalization of oxC_60_ with octadecylamine

100 mg of oxidized fullerenes, dissolved in 50 mL of distilled water, were mixed with a solution of 300 mg of octadecylamine (ODA) in 16.5 mL of EtOH while the pH was set to 7 and the system was stirred for 24 h. The product was collected by centrifugation, washed with ethanol and dried at room temperature (sample denoted as **oxC**_**60**_**-ODA**). The synthesized material presented a yield of approximately of 45% (based on the weighted mass).

### Non covalent immobilization of laccase

Laccase from White rot fungi (10,000 U/mL, WrfL) was purchased from Creative Enzymes (New York, USA). Carbonyldiimidazole (CDI), N-hydrosuccinimidyl ester (NHS), Coomassie Brilliant Blue G-250 (CBB), Bromophenol Blue (BpB), 1-Hydroxybenzotriazole (HBT) and (4-(2-hydroxyethyl)-1-piperazineethanesulfonic acid) (HEPES) were purchased from Sigma. In our standard protocol, 3 mg of oxC_60_-ODA in 5 mL of acetate buffer (0.1 M, pH 4.58) were sonicated for 30 min. Then 1 mL of WrfL was added and the mixture was incubated under stirring for 1 h at 30 °C. The nanomaterial-enzyme conjugates were separated by centrifugation at 6,000 rpm and then were washed three times with buffer solution to remove loosely bound protein. The immobilized WrfL was dried over silica gel and was stored at 4 °C until used.

### Covalent immobilization of laccase via diimide-activated amidation

3 mg of oxC_60_-ODA in 6 mL of distilled water were sonicated for 30 min. Then 1.2 mL of a 10 mg mL^−1^ CDI aqueous solution was added to the above suspension. Under fast stirring, 2.3 mL of a 50 mg mL^−1^ NHS aqueous solution were added quickly and the mixture was incubated for 30 min at 30 °C. The activated nanomaterials were separated by centrifugation at 6,000 rpm and washed three times with HEPES buffer (50 mM, pH 4.58) to remove the excess of CDI. The activated nanomaterials were re-dispersed in 5 mL of HEPES buffer solution. Then, 1 mL of WrfL was added and the mixture was treated as described for non-covalent procedure.

### Dye decolorization

To study the decolorization ability of the immobilized enzymes, 0.1 mg mL^−1^ of immobilized laccase and 1 mM HBT was added to each dye solution (70 μM of CBB and BpB) followed by incubation in a rotary shaker (30 °C and 120 rpm). The solution was sonicated for 3 minutes to achieve full and stable dispersion of the nanomaterial-enzyme conjugates. Samples were taken from each reaction mixture and the decrease in the absorbance at 545 nm was recorded in specific time intervals. The percentage of dye decolorization was calculated as the formula:

$${\rm{decolorization}}( \% )=[({{\rm{A}}}_{{i}}-{{\rm{A}}}_{{t}})/{{\rm{A}}}_{{i}}]\times 100$$

where, A_*i*_: initial absorbance of the dye, A_*t*_: absorbance of the dye at any time interval. Negative controls (reaction mixtures without enzyme) were designed as a reference to compare decolorization percent of treated samples. Each decolorization experiment was performed in triplicate and mean of decolorization percentage was reported.

## Results and Discussion

**Figure 2:**
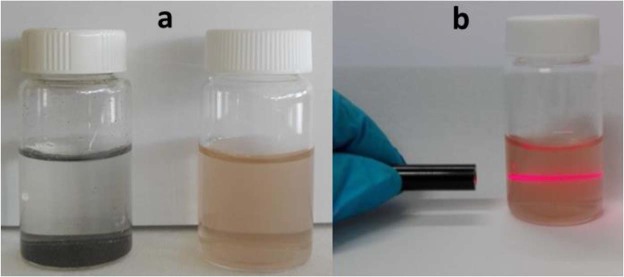
(**a**) Completely insoluble C_60_ molecules in water (left) and water soluble oxidized fullerene (right), (**b**) Tyndall scattering observed when a laser pointer is directed onto an aqueous colloidal dispersion of oxC_60_.

The first indication of successful oxidation came from the solubility behavior of the products estimated at 13 mg/mL (Fig. 2a), a high value compared to other synthetic procedures^60^: The enhanced solubility in water and other polar solvents (like DMSO, 1 mg/mL) can be explained only if a hydrophilic sheath of oxygen-containing groups attached to and surrounding the surface of the fullerene cage is present. Such a high dispersibility in water is expected for polar oxygen-containing groups, distributed more or less homogeneously around the spheroid thus preventing aggregation^63^. Tyndall scattering, observed when the beam of a laser pointer is directed onto an aqueous colloidal dispersion of oxC_60_, provides additional proof for the successful oxidation of fullerenes (as shown in Fig. 2b). This phenomenon is a clear evidence of an excellent dispersibility of C_60_ molecules in aqueous media.

**Figure 3:**
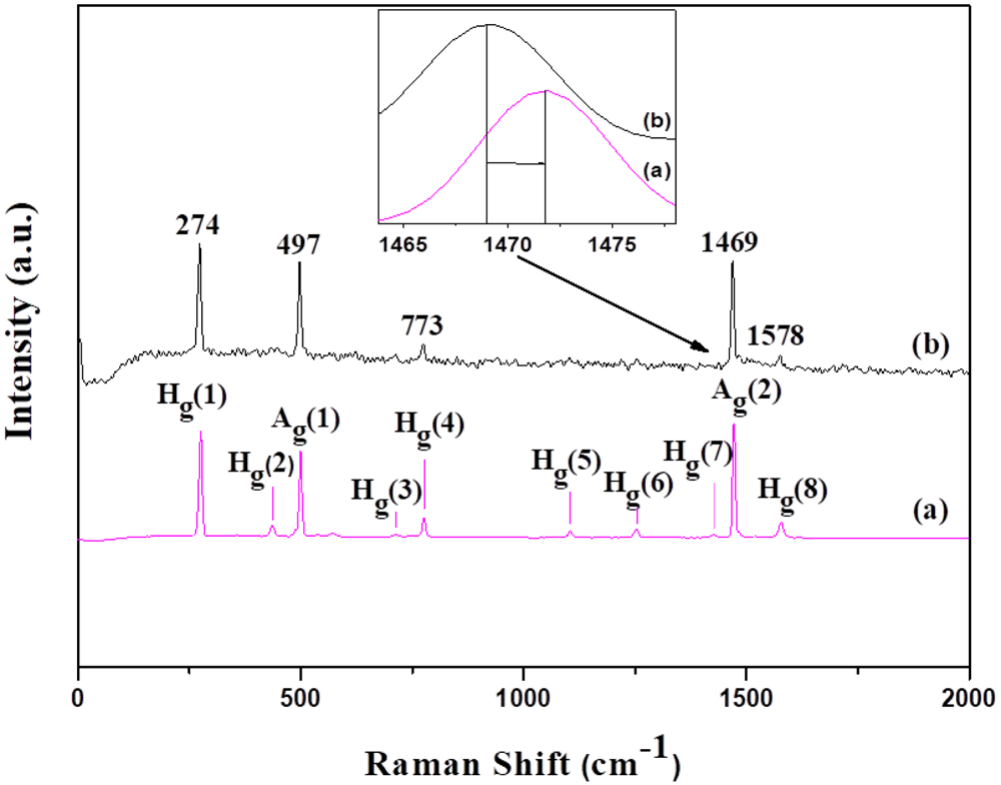
Raman spectra of (**a**) pristine C_60_ and (**b**) oxidized fullerene (oxC_60_).

Vibrational spectroscopy was employed to identify chemical and structural changes as well as to verify the molecular integrity of the C_60_ cage before and after the chemical modification. Figure 3 displays the Raman spectra of C_60_ and of oxC_60_ powder samples. In pristine C_60_ 10 of the 46 vibration modes are Raman active (2A_g_ + 8H_g_) and four (4F_1u_) are infrared active^2,64,65^. The most prominent bands centered at 499 cm^−1^ and 1471 cm^−1^ (Fig. 3a) are assigned to the symmetrical radial breathing motion of the sixty carbon atoms (A_g_(1)) and to the tangential stretching mode of five-fold pentagon carbons (pentagonal pinch mode A_g_(2)), respectively^2,64,65^. The rest of the bands are attributed to the eight H_g_ Raman active modes, distributed between 273 cm^−1^ and 1578 cm^−1^ as indicated in Fig. 3a. After oxidation, the number of active Raman modes decreases with respect to pristine fullerite (Fig. 3b). Only the most intense bands are visible after oxidation, and exhibit a small shift of approximately 2 cm^−1^ with respect to those of C_60_. These changes can be attributed to hindrance of the free rotation motion due to the creation of oxygen-containing functional groups: while the C_60_ molecules in pristine fullerite behave as free rotors at room temperature, the addition of hydroxyl and epoxy/carbonyl groups on the surface of the cage likely leads to the reduction of this rotational movement at ambient temperature possibly due to steric effects or inter-molecular bonding^66^. Importantly, the 1469 cm^−1^ band persists after the oxidation process. This vibration is due to the symmetric A_g_ vibration mode of the spherical framework of the C_60_ cage, thus confirming that the icosahedral structure is intact^67,68^.

**Figure 4:**
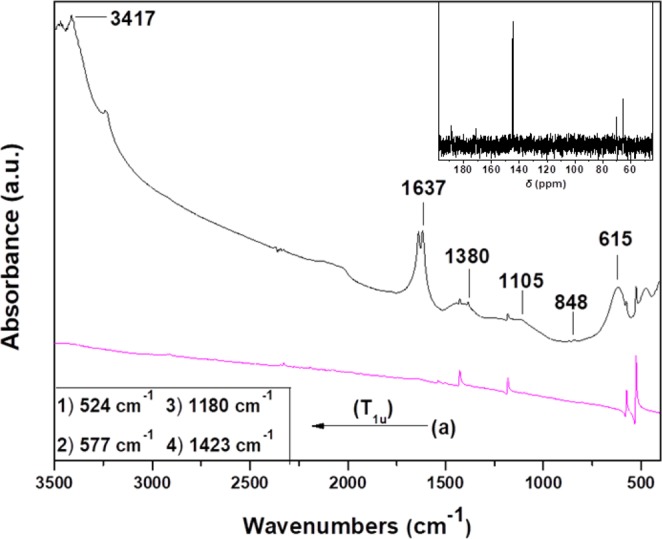
FT-IR spectra of (**a**) pristine (C_60_) and (**b**) oxidized fullerene (oxC_60_). Inset: ^13^C NMR spectrum of oxC_60_ was recorded in D_2_O.

The FTIR spectra of C_60_ and oxC_60_ are presented in Fig. 4. The four IR active vibration modes with F_1u_ symmetry of pristine fullerite (a) are located at wavenumbers of 524, 577, 1180, and 1423 cm^−1^ and assigned to radial displacements of the carbon atoms for the two lowest wavenumber bands and to tangential modes of carbon atoms for the two modes above 1000 cm^−1 ^^69^. The infrared spectrum of the oxidized fullerene (b) reveals the existence of additional peaks compared to C_60_, namely bands at 615 cm^−1^ and 848 cm^−1^, which are assigned to wagging vibrations of hydroxyl groups and in the wavenumber range between 1050 cm^−1^–1470 cm^−1^ three new bands, one centered at 1105 cm^−1^, which is attributed to stretching vibrations of C-O-C ether (epoxide) species^70^, and two located at 1380 cm^−1^ and 1442 cm^−1^ stemming from stretching vibrations of C-OH groups^71^. Finally, the intense band at 1637 cm^−1^ might be due to water bending, which would be in agreement with the increased hydrophilic character of the oxidized fullerene derivatives.

The successful oxidation of C_60_ was also assessed by ^13^C NMR spectroscopy. Specifically, in the ^13^C NMR spectrum of oxC_60_ (Fig. 4 inset, right top), a peak at 144 ppm attributed to the carbon atoms of the cage is shown. Additionally, the peaks at 186 and 171 ppm are assigned to carbon atoms of carbonyl and carboxyl groups, respectively, while the peaks at 71 and 67 ppm are attributed to carbon atoms of epoxy and tertiary alcoholic moieties. Therefore, these findings, which are in line with the FTIR results and the literature^72^, suggest the successful decoration of oxC_60_ with oxygen containing groups, which are mainly epoxy and hydroxyl groups, but also carbonyl and carboxyl moieties are present.

**Figure 5:**
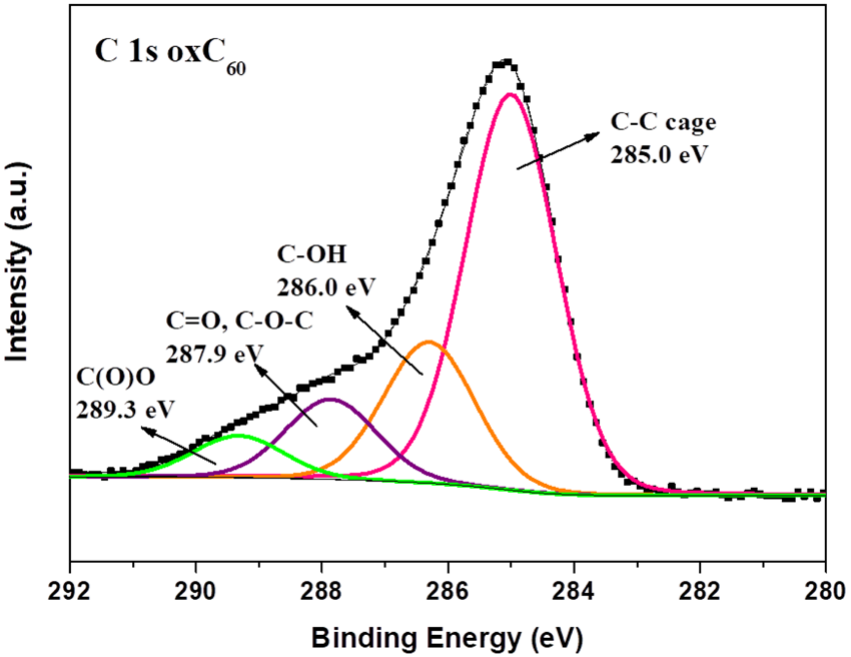
X-ray photoemission spectrum of the C1*s* core-level region of oxidized fullerene (oxC_60_).

X-ray photoelectron spectroscopy (XPS) was applied to attest the presence of oxygen-containing functional groups covalently attached on oxC_60_. XPS is a widely used technique for the chemical characterization of fullerene derivatives since it is not only sensitive to all chemical elements (except H), but also to the local environment surrounding the atoms of that element in a given compound. The C1*s* core level region of the XPS spectrum of oxC_60_, presented in Fig. 5, displays four components at 285.0, 286.0, 287.9 and 289.3 eV. The most intense one at 285.0 eV arises from the carbon-carbon aromatic bonds, and accounts for 60.1% of the total carbon intensity, while rest of the spectral intensity stems from carbon atoms that are involved in heterogeneous bonds. A percentage close to 60% may be expected since the maximum amount of side groups that can be covalently attach to single carbon atoms of the C_60_ cage without any two being adjacent, is 24; this is also the highest number stated for methylization, chlorination or bromination of fullerene^3^ entailing that 36 of the 60 atoms of the carbon cage (*i.e*., exactly 60%) are not bonded to any functional group. The component centered at 286.0 eV is due to carbon atoms forming C-OH bonds and represents 21.6% of the total C1*s* intensity. The contribution at 287.9 eV is assigned to C=O double bonds and/or C-O-C epoxy moieties, and accounts for 12.1% of the total carbon signal. The spectral profile reveals a contribution located at 289.3 eV which represents 6.2% of the total carbon amount. This weak contribution can be accounted for by considering that the strong oxidation treatment probably leads to the creation of a small amount of carboxyl groups. If this small percentage is discarded for the quantitative analysis, then our XPS results indicate that the average oxC_60_ molecule has 22 of the 60 carbon atoms involved in heterogeneous bonds and consists of a C_60_ cage surrounded by 14 hydroxyl groups and by either 4 epoxy moieties or 4 – *m* epoxy moieties and 2 *m* carbonyl oxygens with *m* between 1 and 4 (the number of carbon atoms forming an epoxy moiety is twice the number of epoxy oxygens as each oxygen is linked to two carbons). This yields the average chemical formula for oxC_60_ as C_60_(OH)_14_O_n_ with n between 4 and 8. Taking into account also the ratio between the intensities of the C1*s* and O1*s* photoemission lines (normalized with the respective sensitivity factors), these results confirm the successful oxidation of C_60_ by the creation of functional oxygen groups with a ratio of carbon to oxygen (C/O) equal to 2.2^60^.

**Figure 6:**
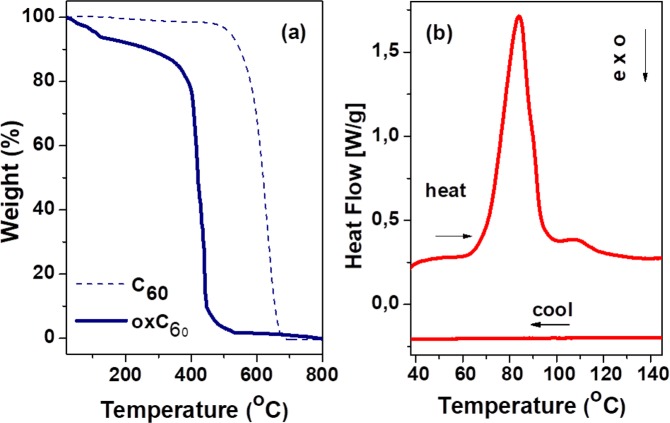
(**a**) TGA curves of pristine and oxidized fullerene. (**b**) DSC thermogram of oxidized fullerene between 40 and 145 °C (heating-cooling cycle).

Further evidence for successful oxidation of C_60_ is provided by thermal decomposition experiments: the required temperature for desorption of functional groups bound to C_60_ is significantly lower than the decomposition temperature of the pure fullerene, enabling also selective removal of the oxygen functional groups in a thermal scan (see below). Figure 6(a)presents the TGA plots of the oxC_60_ sample and of the initial C_60_ material measured with a heating rate of 5 °C/min in air. As evident from the TGA curve of the pristine C_60_ sample, the combustion of fullerite takes place at temperatures between 500 and 700 °C. In the case of oxidized fullerenes, the sample is found to combust at lower temperatures. In fact, the weight loss already starts before 100 °C (due to loss of the epoxy and carbonyl side groups, see below) and progressively continues until a total weight loss is reached when heating the sample up to 700 °C. The main drop in the mass, corresponding to decomposition (combustion) of the oxidized fullerene cages, occurs between 350 and 470 °C, *i.e*. at considerably lower temperature than the decomposition temperature of pure fullerene^73^ due to the presence of hydroxyl groups, as detailed in the following.

Figure 6(b) displays the scanning calorimetry data acquired on as-synthesized oxC_60_ during a heating-cooling cycle between 40 and 145 °C. Upon heating, the oxC_60_ powder displays an irreversible double endothermic transition with onset at 65 °C, a more intense peak just above 80 °C and a minor one above 100 °C. A very similar line shape is obtained by differentiating the TGA data (not shown). As visible in Fig. 6(a), such a double endothermic transition is accompanied by a mass loss of approximately 6.5%, a value consistent with the loss of only the oxygen groups (epoxy and carbonyl) according to the chemical formula C_60_(OH)_14_O_n_ with n between 4 and 5.

**Figure 7:**
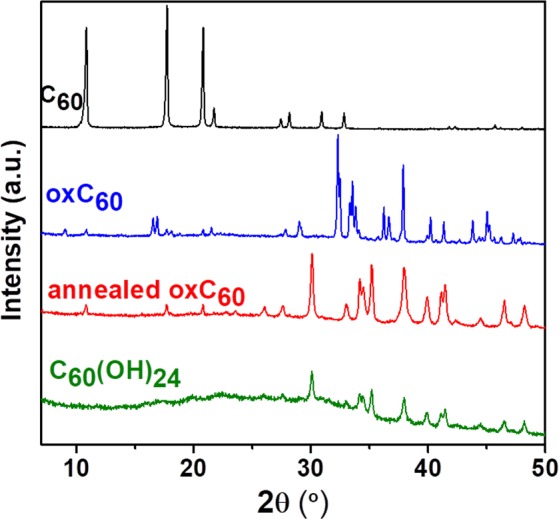
Room-temperature X-ray powder diffraction pattern of oxidized fullerene (oxC_60_), both prior to and after annealing at 100 °C. For comparison, also the XRD patterns of pristine fullerite (C_60_) and of fullerol [C_60_(OH)_24_] are shown (own data).

The confirmation that the endothermal mass loss is due to selective breaking of the oxygen side groups is provided by the analysis of the powder X-ray diffraction results. The room-temperature diffraction pattern of as synthesized oxC_60_ (Fig. 7) indicates that the sample is polycrystalline. The diffraction profile is quite different from that of pristine fullerite (also shown in the same figure for comparison), which confirms the successful functionalization of C_60_ via oxidation^74^. Only traces amount of unreacted C_60_ are present, detectable by the presence of minor diffraction peaks in correspondence to the three main Bragg peaks of pristine fullerite. The pattern of oxC_60_ exhibits the first diffraction peak around 9° in 2θ scale, *i.e*., at a significantly lower angle than the first peak of pristine C_60_ (approximately 11°), indicating that the lattice spacing is larger in oxC_60_ than in fullerite due to the presence of the side groups, which act as steric barriers against denser packing. Upon annealing at temperatures higher than 75 °C, the diffraction pattern of the oxidized fullerene powder changes abruptly, with the appearance of new peaks and the disappearance of all the peaks characteristic of the structure of as-synthesized oxC_60_ (at the same time, the pristine C_60_ peaks become more visible). The resulting spectrum (labeled as “annealed oxC_60_” in Fig. 7) is strongly reminiscent of that of polyhydroxylated fullerenes (fullerol C_60_(OH)_24_) or that of the related derivative C_60_(ONa)_24_^75^, both of which were synthesized following a completely different route than that used here to produce oxC_60_^53,76^. The fact that the diffraction peaks of the oxC_60_ sample warmed to 80 °C or above match those of fullerol indicates that annealing causes selective disruption of the oxygen adducts (epoxy and carbonyl), leading, as a result of the partial decomposition, to the formation of polyhydroxylated fullerenes. An only partial decomposition may be expected since the oxygen adducts are more reactive and therefore more labile than the hydroxyl groups, which in fullerol are lost only above 150 °C^53^. The partial decomposition and the survival of hydroxyl groups also rationalize why the final combustion of the sample occurs at much lower temperatures than for pristine C_60_ (Fig. 6(a)). The observation of a diffraction pattern identical to that of the high-symmetry C_60_(O*X*)_24_ molecules (*X* = H, Na) is a direct confirmation that the quasi-spherical shape of pristine fullerene is retained after oxidation to oxC_60_. The synthesized product is therefore characterized by a symmetric distribution of functional moieties around the carbon skeleton^63^.

**Figure 8:**
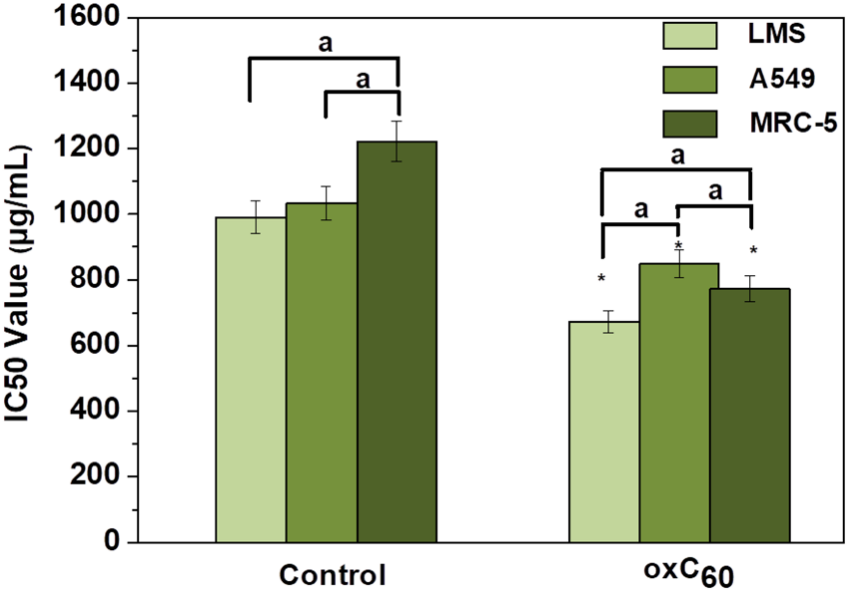
IC_50_ values in μg/mL of oxC_60_ on LMS, A549 and MRC-5 cells. *p < 0.05, statistical significant difference compared to the control (solvent); ^a^p < 0.05, st^a^tistical significant difference between the cell lines within the treatment group.

Very few studies have evaluated the cytotoxic activities of fullerenes. In the present study we evaluated the *in vitro* cytotoxic activity of oxC_60_ against LMS and A549 (cancer cell lines) and MRC-5 (normal cell line). Figure 8 represents the IC_50_ values of the oxidized fullerenes (oxC_60_) in all above mentioned cell lines. It was shown that the oxC_60_ presented high toxicity in all cell lines compared to the control (solvent) (p < 0.05). Also, within the treatment group oxC_60_ showed higher toxicity in LMS cells with an IC_50_ of 670 ± 42 μg/mL. Significant lower toxicity was shown in the other two cell lines with the IC_50_ values to be 850 ± 20 μg/mL and 775 ± 41 μg/mL for A549 and MRC-5, respectively (p < 0.05).

It has been shown previously that C_60_ is able to inhibit the cell growth mainly due to its antioxidative properties^77^ and partly through other mechanisms^78,79^. Also, fullerenes cause cytotoxicity in various cancer cell lines through the molecular events of oxidative stress^80–82^ or lipid peroxidation^83,84^ Here, we have shown that oxC_60_ caused significant cytotoxicity in cancer and normal cell lines. Although the exact mechanism of action is not known, we speculate that oxC_60_ acts in the same way as fullerenes, and possibly through the oxidative stress mechanism.

To confirm the presence of epoxy moieties on the oxC_60_ cage and thus the possibility to further functionalize the oxidized fullerene, an additional experiment was performed in which a primary aliphatic amine (octadecylamine, ODA) was successfully attached through covalent bonding onto the surface of the oxC_60_ molecule. Chemical grafting of the amine end groups via SN_2_ nucleophilic substitution reactions can only take place on the epoxy groups present in the oxidized fullerene. The organophilic character of the produced fullerene derivative resulted in an enhanced solubility in organic solvents including hexane (0.4 mg/mL), toluene (1.5 mg/mL), and chloroform (1.5 mg/mL), which provides the primary evidence for the covalent functionalization of oxC_60_ with ODA.

**Figure 9:**
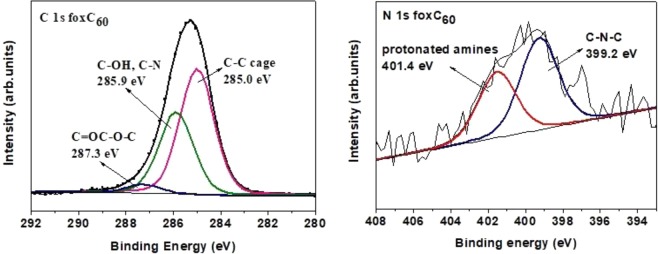
X-ray photoemission spectrum of the C1*s* (left) and N1*s* (right) core level regions of functionalized oxidized fullerene (oxC_60_–ODA).

XPS and FTIR were employed to verify the covalent bonding of ODA on the surface of oxC_60_. After functionalization with ODA, XPS measurements revealed the presence of new components stemming from the formation of covalent carbon-nitrogen bonds at the epoxy sites. More specifically, the analysis of the C1*s* XPS spectrum of oxC_60_–ODA (Fig. 9 left) allows singling out the characteristic component due to carbons involved in the fullerene cage as well the C-C chain of the –ODA at 285.0 eV contributing with 58.2% to the total C1*s* intensity. The relative spectral weight of the feature at 285.9 eV is significantly larger than in the oxC_60_ case (Fig. 5), changing from 21.6% before to 37.9% after the functionalization. This change is due to the creation of covalent C-N bonds linking the organic chains to the carbon cage structure, as the binding energies values for carbon atoms linked to amine or hydroxyl groups are very similar^62^. Lastly, a weak component centered at 287.3 eV and representing 3.9% of the total C1*s* intensity is attributed to carbonyl moieties, since the contribution of the epoxy oxygens is absent due to the creation of the C-N-C bridges of the organo-modified fullerene derivative. Very intriguing and a clear evidence for the integrity of fullerene molecules, is the total absence of the spectral signature of carboxyl groups in the C1*s* spectrum of oxC_60_-ODA (Fig. 9, left panel), unlike for the oxidized fullerene (Fig. 5). Thus the carboxyl groups, which were created (during the acid treatment) due to the breakage of a very small amount of fullerene cages, are absent after organic functionalization. This implies that these formations did not take part in the reaction as soon as carboxylic groups can react with amines under specific conditions and not in ambient conditions by which the experiment took place. A possible explanation for this is that the tiny amount of fullerenes possessing carboxylic moieties keep their hydrophilic character, and are thus removed from the reaction products upon washing with ethanol and water during the synthetic procedure. This provides a means to purify the oxC_60_-ODA derivative.

Additional information on the type of interaction of ODA with the oxidized fullerenes comes from the N1*s* core level region of the photoelectron spectrum (Fig. 9, right-hand panel). The N1*s* line can be modeled with two main components centered at 399.2 eV and 401.3 eV binding energy, which correspond to the creation of the epoxy amine bond (C-N-C)^85–87^ and to protonated amines of the ODA moieties, respectively^88^. This entails that some of the ODA moieties are not covalently bonded to the fullerene cage, but are instead weakly bound to the sample (possibly via the formation of hydrogen bonds with the oxygen-containing groups of oxC_60_). The carbon to nitrogen ratio is estimated at 20.2 showing that each oxidized fullerene molecule is surrounded by several (more than a dozen) ODA moieties. The carboxylated carbon formations, which are created (during the acid treatment) due to the breakage of a very small amount of fullerene cages is absent after the organic functionalization. For this reason by the organic functionalization of ox-C_60_ we can impart new properties to the hydrophilic fullerenes, which is very important for specific applications as well clear the rest of the carbon impurities, which are created from the strong oxidation and destroy the ball-shape of the C_60_.

**Figure 10:**
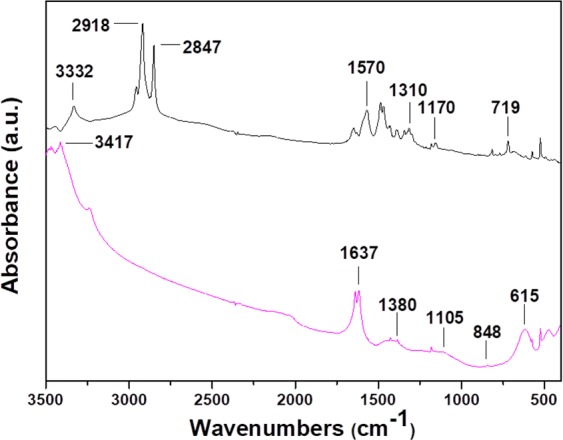
FTIR spectrum of oxidized C_60_ (oxC_60_) (black line) and functionalized oxidized C_60_ (oxC_60_-ODA) (purple line).

The successful incorporation of ODA and the creation of a new fullerene derivative were further confirmed by FT-IR spectroscopy, as shown in Fig. 10. The spectrum of oxC_60_–ODA shows absorption bands, which are absent in the spectrum of pristine C_60_ (compare with Fig. 4). More specifically, for the functionalized fullerene we observe a band at 718 cm^−1^, which is attributed to the wagging vibration of N-H stemming from non-covalently bonded ODA. The absorption bands at 1570 cm^−1^ (in plane-deformation) and 3332 cm^−1^ (stretching) are similarly assigned to vibrations of NH_2_ groups, while C-N stretching vibrations are observed at 1170 cm^−1^ and 1310 cm^−1^. Moreover, the peak at 1105 cm^−1^ due to epoxide vibrations disappears upon functionalization, indicating that the primary amines of ODA have reacted with the epoxide groups of the oxC_60_. These results together confirm the initial presence of epoxy oxygens in oxC_60_. Finally, the bands at 2847 and 2918 cm^−1^ are attributed to symmetric and asymmetric vibrations of -CH_2_-and –CH_3_ (alkyl groups), respectively, indicative of the presence of aliphatic hydrocarbon chains of ODA moieties attached to the carbon cage^89^.

### Dye decolorization

**Figure 11:**
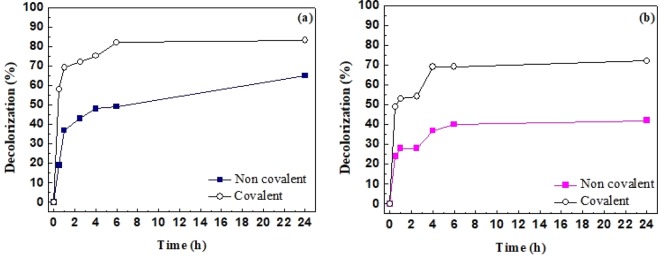
Decolorization of (**a**) CBB and (**b**) BpB by immobilized WrfL on oxC_60_-ODA (In all cases, the standard deviation was <3%).

The synthesized nanomaterial oxC60-ODA was used as matrix for the covalent and non-covalent immobilization of laccase from White rot fungi (WrfL), and its further application for the decolorization of two synthetic dyes of industrial and biotechnological interest, Coomassie Brilliant Blue G-250 (CBB) and Bromophenol Blue (BpB). Laccases are known to be capable of catalyzing the oxidation of synthetic dyes and hold potential for applications in sensing and bioremediation of industrial effluents^90,91^. The mediator hydroxybenzotriazole (HBT) was used to facilitate the decolorization of the chosen dyes. HBT acts as a sort of an electron bridge between the enzyme and the substrate. Firstly, HBT is oxidized by laccase, diffuses away from the enzymatic pocket and in a next step oxidizes other molecules, extending, this way, the range of substrates that can be efficiently catalyzed by laccase, and thus increasing its catalytic activity^92^. The decolorization efficiency of the immobilized laccase against CBB and BpB is presented in Fig. 11. As seen, in all cases studied, both covalent and non-covalent immobilized laccase appeared to efficiently decolorize the chosen dyes to a high extent. More specific, covalently immobilized WrfL presents high efficiency, since the decolorization rate for CBB and BpB reached up to 60 and 50%, respectively, even after 0.5 h of incubation. The results indicate that this novel nanobiocalytic system shows great efficiency for dye degradation, even higher than other immobilized laccases reported previously^93,94^. The beneficial impact of different carbon-based nanostructures on the catalytic characteristics of enzymes has already been demonstrated^92,95^. The use of ODA for the functionalization of oxC_60_ and the targeted immobilization of WrfL increases the space between the nanomaterial and the protein, reducing in this way any undesired interactions between them^95^. Moreover, any substrate diffusion limitations are minimized, resulting in high catalytic activity for dye decolorization. The results indicate that oxC_60_-ODA can be excellent support for enzyme immobilization for use in applications of biotechnological and industrial interest.

## Conclusions

A combination of characterization techniques was applied in order to illustrate the successful chemical oxidation of fullerene (C_60_) into a highly oxidized analogue (oxC_60_) by means of a variant of Staudenmaier’s method, an easy oxidation protocol up to now extensively used for the chemical oxidation of graphite. This oxidation leads to the formation of a highly soluble fullerene derivative (that could be called “fullerene oxide” similar to graphene oxide) through the creation of oxygen-containing functional polar groups on the surface of C_60_, while retaining its spherical structure and represents a very crucial step for the utilization of the entire fullerene family in applications such as medicinal chemistry and biochemistry, which require solubility in various polar solvents. Apart from its simplicity, the main advantage of this method compared to others applied so far for the production of soluble C_60_ derivatives, arises from the creation of epoxy groups on the surface of the fullerene. A novelty of our synthetic approach is that it produces hydrophilic C_60_ molecules decorated with epoxy moieties in high yield but at low cost. The presence of these groups allows for further functionalization and thus for the creation of new hydrophilic fullerene derivatives without the need of high temperatures and complicated synthetic reactions. In fact, the epoxy groups can be readily modified via ring-opening reactions under various conditions. As representative example, a primary aliphatic amine (octadecylamine) was successfully attached through covalent bonding. The proposed method represents a novel simple, versatile and reproducible approach for the controllable production of various well-defined and stable fullerene derivatives by exploiting the well-established carbon chemistry.
